# Risk factors for mental health and barriers to mental health care amongst Somalis in Europe: A mixed methods systematic review

**DOI:** 10.1192/j.eurpsy.2026.10986

**Published:** 2026-07-31

**Authors:** M. Karshe, M. Alkasaby

**Affiliations:** London School of Hygiene & Tropical Medicine, London, United Kingdom

## Abstract

**Introduction:**

Ethnic minorities across Europe experience persistent mental health disparities, having higher rates of psychiatric conditions and lower access to care. However, the issues faced by these groups are not always congruent. By focusing on Somalis in particular, this review explores how specific factors may interact and overlap for one ethnic group. Somalis are largely homogenous, sharing common cultural and religious beliefs and often facing similar experiences of conflict, migration and marginalisation. Examining both risk factors and barriers to care allows for a more integrated understanding of these challenges, highlighting their overlap in a shared sociocultural context.

**Objectives:**

This review aims to identify the risk factors associated with mental health issues in the European Somali population, explore barriers to their access to mental health services and examine how migration-related experiences and other factors influence mental health and service utilisation.

**Methods:**

This review is a mixed-methods systematic review following the PRISMA guidelines and the Joan Briggs Institute guidelines for mixed methods reviews. We conducted a comprehensive search of five databases, Medline, Embase, PsycINFO, Global Health and CINAHL, from the inception dates to July 24^th^ 2025, for peer-reviewed records. Keywords included Somali, mental health, risk factors and barriers. Exclusion criteria included studies undertaken in countries outside of Europe, articles not written in English and studies that did not present exclusive or disaggregated results for Somalis. Articles underwent two-stage screening against the rigorous inclusion and exclusion criteria. Data was extracted using standardised templates, and quality appraisal was undertaken using the mixed methods appraisal tool. Data was synthesised using a convergent, integrated approach.

**Results:**

Twenty-five studies were included (13 quantitative, 10 qualitative and 2 mixed methods). Key risk factors identified included traumatic experiences, social networks and residential instability. Barriers to accessing care included stigma, alternative cultural beliefs and coping strategies, and mistrust of services. Findings varied across genders and ages, with Somali women being less aware of the services available and less likely to utilise services than men. Policy-level factors were seldom addressed in the evidence, highlighting a gap in the current evidence.

**Conclusions:**

Addressing mental health inequalities requires action across multiple levels, including individual, social and structural levels. Findings of this review support the need for culturally informed and responsive mental health care and policies that tackle the intersectional risks individuals from minority groups face.

**Disclosure of Interest:**

None Declared

